# To drain or not to drain in minimal invasive ventral hernia surgery

**DOI:** 10.1007/s00423-025-03668-x

**Published:** 2025-03-11

**Authors:** Stella Wilters, Fadl Alfarawan, Catharina Fahrenkrog, Maximilian Bockhorn, Nader El-Sourani

**Affiliations:** 1 Department for General – and Visceral Surgery, Klinikum Oldenburg AöR, Rahel-Straus-Straße 10, 26133 Oldenburg, Germany; 2 Carl-Von-Ossietzky University Oldenburg, Ammerländer Heerstraße 114-118, 26129 Oldenburg, Germany; 3 Department of General, Visceral and Transplantation Surgery, University Hospital Münster, Waldeyer Straße 1, 48147 Münster, Germany

**Keywords:** Etep, Drain, Ventral hernia repair, SSI, SSO, Seroma

## Abstract

**Purpose:**

Despite the high prevalence of ventral hernias worldwide, intraoperative drain placement remains a controversial topic. The benefit in reducing postoperative complications has not yet been clearly demonstrated. This study investigates whether a drain prevents postoperative complications after minimally invasive ventral hernia repair using the extended-totally-extraperitoneal-(eTEP)-technique.

**Methods:**

This monocentric, retrospective cohort study included all patients who underwent eTEP between 2019 and 2024. Two comparison groups were formed (54 patients with drain,106 patients without) and analysed for potential differences.

**Results:**

There were no significant sociodemographic or clinical differences between the study groups. The defect size was larger in the drain group (drain: 13 cm^2^ (64,5) †, no-drain: 6,5 cm^2^ (21) †, *p* = 0,025). There were no significant differences regarding frequency of postoperative complications (drain: 13%, no-drain: 8,5%, *p* = 0,373), surgical site infections (SSI) (drain: 0%, no-Drain: 1,9%, *p* = 0,550), and surgical site occurrences (SSO) (drain: 13%, no-Drain: 4,7%, *p* = 0,108). A subgroup analysis showed that robotically operated patients were more frequently provided with drains (rob: 30 (47,6%), lap: 24 (24,7%), *p* = 0,003), had larger defect sizes (rob: 28 cm^2^ (72)†, lap: 6 cm^2^ (9,87)†, p < 0,001), and received Transversus-abdominis-releases (TAR) more often (rob: 14 (22,2%), lap: 5 (5,2%), *p* = 0,001).

**Conclusion:**

We found no significant differences between patients with and without drains after eTEP regarding the frequency of postoperative complications, SSOs and SSIs. Our findings do not suggest nor refute that wound drains prevent postoperative complications.

**Supplementary Information:**

The online version contains supplementary material available at 10.1007/s00423-025-03668-x.

## Introduction

Ventral hernias of the abdomen are among the most common pathologies encountered in surgery by general and visceral surgeons [1]. Primary ventral hernias occur in approximately 20% of all adults, while secondary hernias develop in 30% of all cases following abdominal incisions near the midline [2]. Specifically, ventral hernias are defects in the abdominal wall fascia that are neither hiatal nor inguinal in origin [3]. It is estimated that over 6 million patients worldwide undergo ventral hernioplasty each year [1]. In many cases, the placement of a biocompatible mesh is necessary to support the abdominal wall [4].

Belyansky et al. described a novel approach to laparoscopic retro-muscular hernioplasty using the "enhanced-view totally extraperitoneal" (eTEP)-technique in 2018 [5]. This method combines the advantages of open retro-muscular repair with those of minimally invasive surgery by placing the mesh in a sublay technique between the muscle and the rectus sheath. While this technique offers benefits such as superior anatomical positioning of the mesh without fixation and reduced contact with abdominal organs, it is technically more demanding than onlay or underlay procedures [6–8]. Fluid collections like seromas or hematomas may occur in the prepared retro-muscular space, potentially impairing mesh integration and leading to postoperative infections. Such complications can prolong postoperative recovery and also increase healthcare system costs [9]. Nevertheless, the sublay technique is now considered a standard method and is superior to other mesh placements and surgical techniques like intraperitoneal onlay mesh (IPOM), particularly regarding recurrences or postoperative complications [10–14].

To reduce postoperative fluid collections and thereby preventing seroma and hematoma formation, wound drains are commonly used in visceral surgery [1]. A meta-analysis by Marcolin et al. demonstrated that using drains during hernioplasties with retro-muscular mesh placement was associated with a significantly lower rate of postoperative seromas [1]. Similarly, Miller et al. reported that drains reduced the likelihood SSOs after hernioplasty [15].

On the other hand, there is data indicating that drains do not effectively prevent fluid collections: a randomized controlled trial published by Willemin et al. in 2022 found no significant difference between patients with and without drains after open hernioplasty regarding postoperative fluid collections [16]. The authors concluded that routine usage of drains after hernioplasty is not justified.

A meta-analysis published by Mohamedahmed et al. in 2023 observed that patients who received a drain after robotic, laparoscopic or open hernioplasty showed a significantly higher rate of SSI compared to those without drains [3]. Additionally, the authors found no demonstrable preventive effect of drains on seroma formation.

Overall, despite the prevalence of ventral hernias in the global population, placing a drain after hernioplasty remains a controversial topic in surgery because their benefit in reducing postoperative complications has not been clearly established [1]. Therefore, this study aims to investigate whether placing a wound drain after laparoscopic or robot-assisted eTEP hernioplasty effectively prevents surgical complications to contribute to optimizing perioperative care for patients and to provide greater clarity for clinical decision-making.

## Methods

### Study design and patients

This study is a monocentric, retrospective cohort study conducted at the University Hospital for General and Visceral Surgery at Klinikum Oldenburg based on a prospective database that has been maintained since 2019 documenting all patients receiving ventral hernia repair. The study included and retrospectively analysed all patients with ventral hernias who underwent a minimally invasive eTEP procedure between 2019 and 2024. All surgeries during this period were performed by the same surgeon. Using the hospital information system, sociodemographic and clinical data were retrospectively collected from the corresponding patient records and integrated into the dataset.

The cohort was divided into two study groups based on whether a drain was placed or not. The decision to use a drain was made intraoperatively, taking into account the patient's use of anticoagulants and the presence of a diffuse bleeding tendency.

The occurrence of postoperative complications like haematomas, seromas or infections in the surgical area within 30 days post-operation constituted the primary outcome of this study. To assess this, all patients were invited to follow-up appointments 30 days after their surgery. Postoperative complications that occurred before the follow-up appointment were documented and treated either during the hospital stay related to the surgery or during an earlier consultation by the patient. Data collection was conducted with the approval of the relevant ethics committee at the University of Oldenburg (AZ2024-093).

### Inclusion and exclusion criteria

**Fig. 1:**
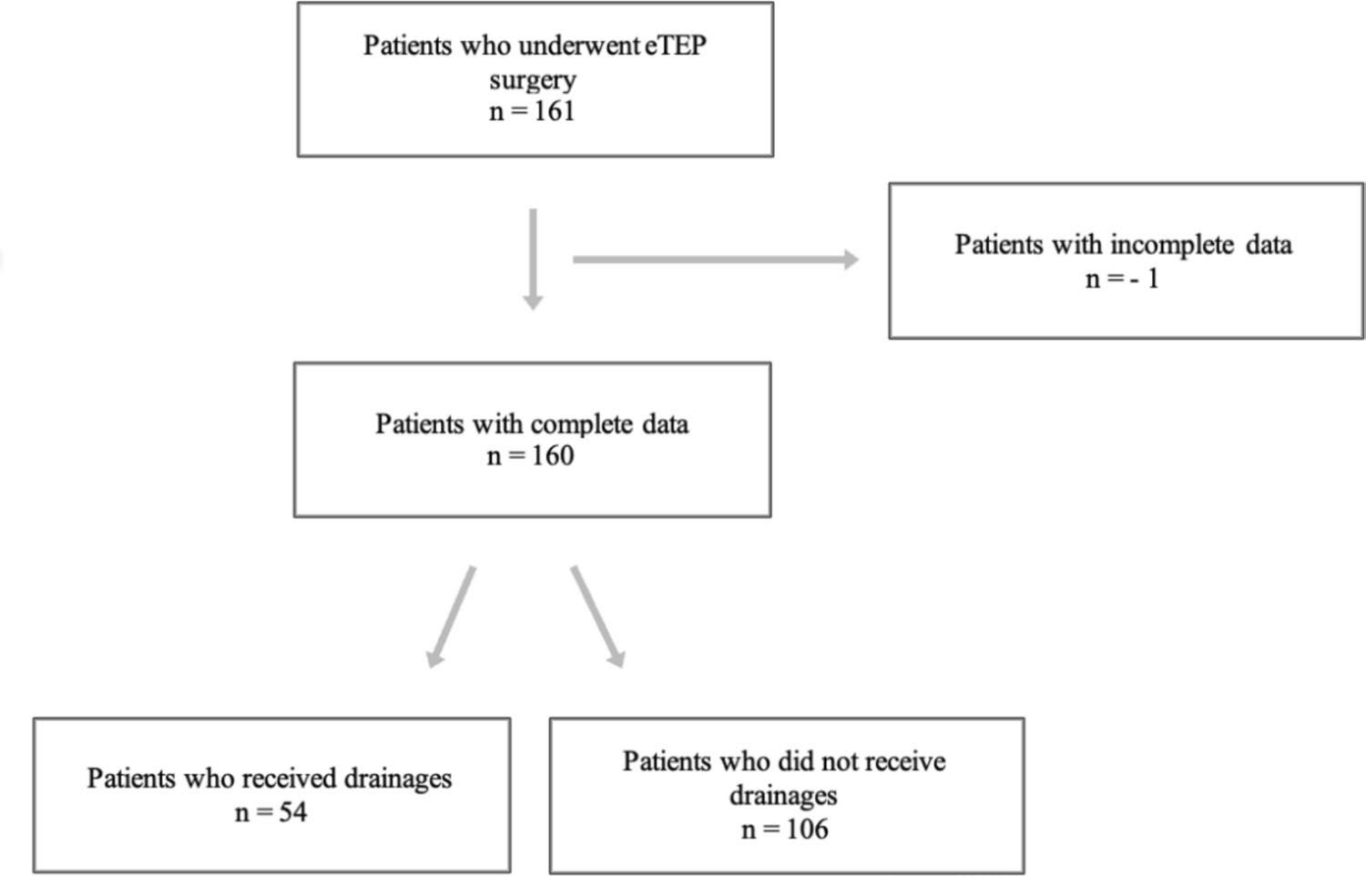
Flow-Chart showing the inclusion and exclusion of patients

Figure 1 shows the inclusion and exclusion of study participants. All patients who were of legal age (18 years and older) and underwent hernioplasty using the eTEP technique were included in the study, regardless of whether they were operated laparoscopically or robotically (*n* = 161). One patient was excluded due to incomplete data. In total, 160 patients met the inclusion criteria for this study.

### Variables and endpoints

The collected demographic data included gender, age, body mass index (BMI), and type of hernia (primary or incisional). Additionally, data on potential risk factors that could influence the outcome were gathered, such as pre-existing conditions of the cardiovascular system (myocardial infarction, atherosclerosis, coronary heart disease, hypertension, heart failure, atrial fibrillation, valve insufficiencies, valve stenoses), lung conditions such as Chronic Obstructive Lung Disease (COPD) or asthma, diabetes, obesity (BMI > 30 kg/m^2^), smoking status, liver insufficiency, and the use of anticoagulants.

The perioperative risk of patients was assessed using the American-Society-of-Anaesthesiologists (ASA) classification. Furthermore, the location of all hernias was described and recorded using the European-Hernia-Society-(EHS)-classification. In patients with more than one hernia, each location was considered a separate hernia and documented accordingly. Consequently, the total number of documented hernias was higher than the number of patients. Additionally, the following operative data were collected: surgical procedure (robotic or laparoscopic), duration of surgery in minutes, size of the defect in cm^2^, size of the mesh in cm^2^, performance of TAR, intraoperative complications, received erythrocyte concentrates and placed drains.

Ultimately, postoperative findings included: postoperative complications, wound-specific complications such as SSO and SSI, surgical site occurrences requiring intervention (SSOPI), length of hospital stay in days, recurrences, any postoperative antibiotic therapy received and the Clavien-Dindo-Score. SSOs were defined as all non-infectious wound-specific complications such as haematomas, seromas, haematoma-seromas, wound healing disorders or delays, wound dehiscences, early recurrences and necroses. All deep or superficial wound infections were counted as SSIs. If SSOs or SSIs required an invasive intervention such as reoperation, puncture or suturing, they were additionally categorised as SSOPI.

### Surgical technique—laparoscopic eTEP

**Fig. 2:**
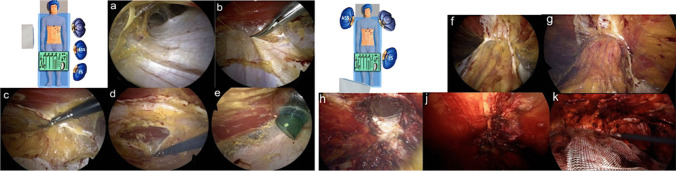
Laparoscopic eTEP **a**) Blunt dissection of the retro muscular space; **b**) Transection of the posterior fascial layer; **c**) Preperitoneal dissection behind the linea alba; **d**) Transection of the contralateral posterior fascial layer and opening of the retro rectal space; **e**) Placement of the camera trocar in the contralateral retro muscular space; f) Cranio caudal view of both retro-muscular and preperitoneal space; g) Cranial depiction of the hernia; h) Repositioning of the hernia sac and content; j) Continuous closure of the defect; k) Placement of the mesh

The following describes eTEP surgery of a lower midline hernia. The first 11-mm optical trocar is inserted into the retro muscular space. The trocar is placed along the rib margins, approximately on the medio clavicular line. After blunt dissection, the retro muscular space is insufflated with CO2 at 20 mmHg (Fig. 2a). Next, two additional 5-mm working trocars are inserted along the linea semilunaris. Dissection progresses toward the medial edge of the posterior layer of the rectus sheath, exposing the fusion between the anterior and posterior layers. A crossover is performed to create a connection between the preperitoneal space behind the linea alba and both retro muscular spaces (Fig. 2b-d). A fourth 11-mm trocar is placed on the opposite side of the midline (Fig. 2e), and the optic is switched to this trocar for improved visibility. Dissection continues from cranial to caudal. The hernia sac is identified, and its contents are reduced. Dissection is extended at least 5 cm below the hernia defect. (Fig. 2f-h). The size of the hernia is measured, and the defect is closed. The linea alba is reconstructed using a long-term absorbable, self-fixating Stratafix suture (0) (Fig. 2j). Any defects in the posterior fascial layer are closed with 2–0 Stratafix sutures. After measuring the prepared space, a biocompatible mesh (Soft-Mesh BD Bard ®) is inserted. If necessary, a drain may be placed. Finally, while ensuring correct positioning of the mesh the gas is released from the retro muscular space (Fig. 2k).

### Surgical technique—robotic eTEP

The first 11-mm optical assist trocar is inserted into the retro muscular space. The trocar is placed along the rib margins, approximately on the left medio clavicular line. The retro muscular space is insufflated with CO2 at 20 mmHg, allowing for further blunt dissection while preserving the lateral neurovascular bundle. Next, three additional 8-mm robotic trocars are inserted into the retro muscular space along the linea semilunaris at 6 cm intervals and 2 cm away from bony structures.

**Fig. 3:**
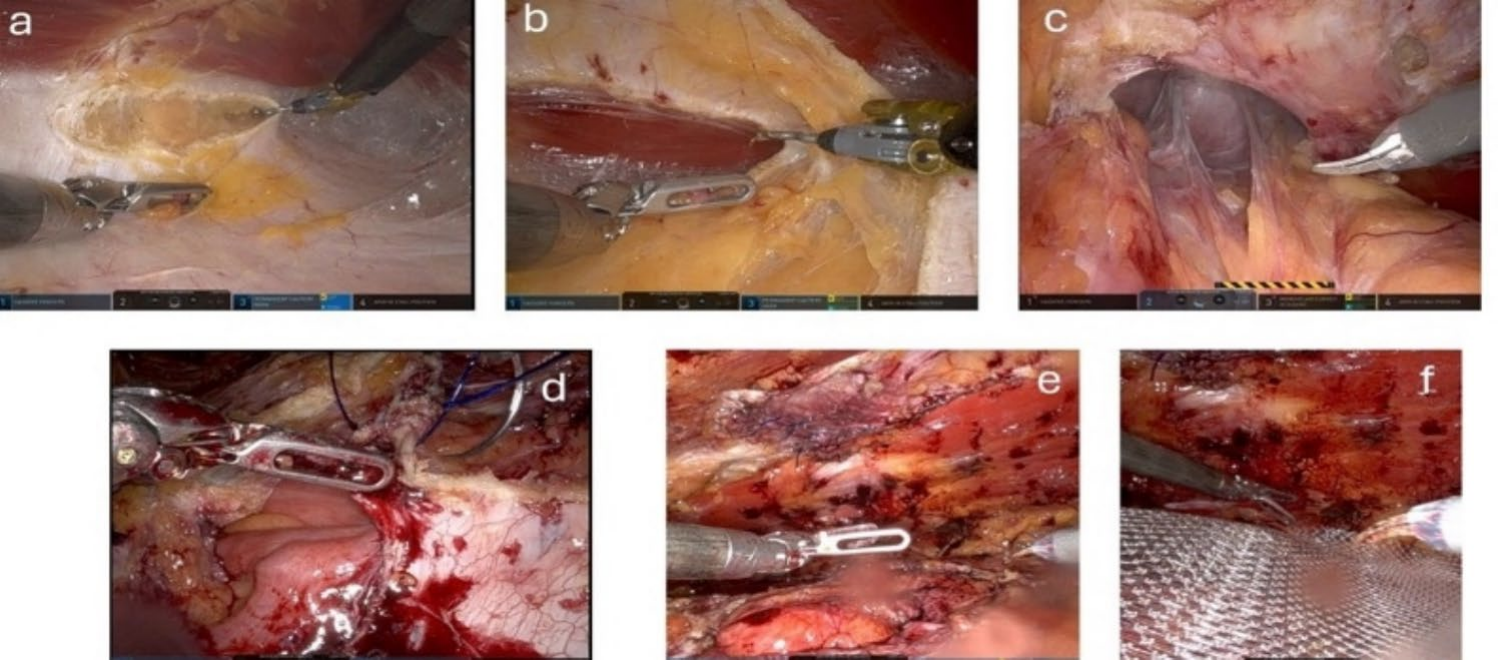
Robotic eTEP **a**) Transection of the posterior fascial layer on the left side; **b**) Preperitoneal dissection behind the linea alba and transection of the contralateral posterior fascial layer, opening of the right sided retro muscular space; **c**) Repositioning of the hernia sac; **d**) Closure of the hernia defect; **e**) closed defect; **f**) Placement of the mesh

The procedure of robotic eTEP follows the main steps as described for laparoscopic eTEP: 1. Completing the dissection of the retro muscular space. 2. Visualisation of the medial edge of the posterior layer of the rectus sheath and exposing the fusion between the anterior and posterior layers. 3. Incision of the posterior layer of the rectus sheath on the ipsilateral side (Fig. 3a). 4. Preperitoneal dissection behind the linea alba, opening the retro muscular space behind the contralateral musculus rectus abdominis (Fig. 3b). 5. Repositioning of the hernia sac (Fig. 3c). 6. Reconstruction of the Linea alba along with the defect and the posterior layer of the rectus sheath (Fig. 3d). 7. Expanding the retro muscular space behind the contralateral musculus rectus abdominis (Fig. 3e). 8. Placement of the mesh (Soft-Mesh BD Bard ®) (Fig. 3f). 9. Placement of Drain if necessary.

### Surgical technique—transversus-abdominis-release

In cases of large or complex defects, a TAR can be performed during the eTEP operation [17]. Large hernias are defined as defects wider than 10 cm or fascial defects affecting more than 25% of the abdominal wall. TAR is also an appropriate method for abdominal wall repair in hernias of the lateral abdominal wall, particularly in the lumbar or flank regions.

Initially, the posterior fascia of the internal oblique muscle is incised medially to the neurovascular bundle to expose the transversus abdominis muscle [18]. Subsequently, the fibres of the transversus abdominis muscle that insert into the posterior rectus sheath are transected. The transversalis fascia along with peritoneum is carefully detached laterally and caudally from the muscle. Following this, the actual hernioplasty can proceed.

### Statistical analysis

All analyses were conducted using SPSS (Version 29.0). All categorical variables were reported with absolute and relative frequencies, while all continuous variables were presented with means and standard deviation if they were normally distributed, and with medians and interquartile ranges if they were not normally distributed. The distribution of all continuous variables within the comparison groups was tested using the Shapiro–Wilk test. For the comparison of the two study groups, a chi-squared test was performed for categorical variables. In cases where expected cell frequencies were less than 5, Fisher's exact test was employed. An unpaired t-test was used for continuous variables that were normally distributed to determine significant differences between the study groups. For non-normally distributed continuous variables, the Mann–Whitney-U-test was applied. In order to adjust for potential confounders multivariable regressions were used: Logistic regression for binary outcomes, linear regression for duration of surgery, and Poisson regression for length of stay in days beyond the first day. A p-value of ≤ 0,05 was considered statistically significant. No adjustment for multiple testing was made.

In a secondary analysis, robotic-eTEP patients and laparoscopic-eTEP patients were selected as comparison groups to investigate whether certain variables influenced the results of the primary analysis. Subgroup analyses were also conducted for patients with EHS L1-L4 hernias, robotically operated patients, laparoscopically operated patients, patients with incisional hernias, and patients with primary hernias.

## Results

### Patient characteristics

In total, 160 patients with abdominal wall hernias underwent eTEP surgery between July 2019 and June 2024. Of these patients, 54 were provided with a wound drain intraoperatively, while 106 patients did not receive a drain. No significant differences were observed in gender distribution between the groups. The average age in both groups was also similar (Drain: 52,72 years (range: 30–79 years), no-drain: 53,27 years (range: 18–86 years), *p* = 0,483). The BMI was comparable in both groups as well (drain: 30,5 kg/m^2^ (11,5) †, no-drain: 30 kg/m^2^ (8) †, *p* = 0,775). ASA score II was the most prevalent among patients in both groups (drain: 36 (66,7%), no-drain: 66 (62,3%). There were no significant differences regarding the distribution of ASA classification between the two comparison groups. The frequency of risk factors in the form of pre-existing conditions wasalso similar in both groups. Further information can be found in Table 1.

**Table 1:** Patient Demographics

	Total (*n* = 160)	Drain (*n* = 54)	No-Drain (*n* = 106)	*p value*
**Sex, n (%)**				
Male	95 (59,4)	30 (55,6)	65 (61,3)	0,483
Female	65 (40,6)	24 (44,4)	41 (38,7)	
Age in years, mean (SD)	53,09 (± 13,44) *	52,72 (± 12,96) *	53,27 (± 13,74) *	0,807
BMI in kg/m^2^, median (IQR)	30 (9) †	30,5 (11,5) †	30 (8) †	0,775
ASA-Score, n (%)				
1	14 (8,8)	3 (5,6)	11 (10,4)	0,386
2	102 (63,7)	36 (66,7)	66 (62,3)	0,584
3	44 (27,5)	15 (27,8)	29 (27,4)	0,955
4	0 (0)	0 (0)	0 (0)	/
5	0 (0)	0 (0)	0 (0)	/
Preexisting condition: Cardiovascular system, n (%)	76 (47,5)	25 (46,3)	51 (48,1)	0,828
Preexisting condition: Lung, n (%)	18 (11,3)	7 (13)	11 (10,4)	0,625
Obesity, n (%)	80 (50)	28 (51,9)	52 (49,1)	0,738
Diabetes, n (%)	20 (12,5)	9 (16,7)	11 (10,4)	0,255
Smoking, n (%)	38 (23,8)	12 (22,2)	26 (24,5)	0,746
Liver insufficiency, n (%)	1 (0,6)	0 (0)	1 (0,9)	1,000
Type of hernia, n (%)				
Primary	104 (65)	33 (61,1)	71 (67)	0,462
Incisional	56 (35)	21 (28,9)	35 (33)	
Use of anticoagulants, n (%)	22 (13,8)	6 (11,1)	16 (15,1)	0,489

* = mean + SD; † = median + IQR

### Hernia types—EHS-classification

In both groups, umbilical hernias classified as EHS-M3 were the most frequently represented (drain: 49 (53,8%), no-drain: 81 (52,3%)). Overall, there were no significant differences regarding the EHS classifications between the study groups (see Table 2).

**Table 2:** EHS classification

	Total (*n* = 246)	Drain (*n* = 91)	No-Drain (*n* = 155)	*p value*
European Hernia Society Classification, n (%)				0,945
M1	5 (2,03)	1 (1,1)	4 (2,6)	0,654
M2	75 (30,5)	29 (31,9)	46 (29,7)	0,823
M3	130 (52,9)	49 (53,8)	81 (52,3)	0,810
M4	23 (9,4)	9 (9,9)	14 (9)	1,000
M5	4 (1,6)	1 (1,1)	3 (1,9)	1,000
L1	1 (0,4)	0 (0)	1 (0,6)	1,000
L2	5 (2,)	2 (2,2)	3 (1,9)	1,000
L3	3 (1,2)	0 (0)	3 (1,9)	0,298
L4	0 (0)	0 (0)	0 (0)	-

### Operative findings

The results regarding operative data are presented in Table 3. The duration of surgery in minutes was similar in both groups and did not differ significantly (*p* = 0,082). The frequency of TAR was also comparable between the two groups (*p* = 0,761). No complications were observed intraoperatively in both study groups. Patients who received a drain underwent robotic surgery significantly more often than those who did not receive a drain (drain: 30 (55,56%), no-drain: 33 (31,13%), *p* = 0,003). The average defect size was significantly larger in the drain group compared to the no-drain group (drain: 13 cm^2^ (64,5) †, no-drain: 6,5 cm^2^ (21) †, *p* = 0,025).

**Table 3:** Operative Findings

		Total (*n* = 160)	Drain (*n* = 54)	No-Drain (*n* = 106)	*p value*
Surgical procedure, n (%)					
Robotic Laparoscopic		63 (39,4) 97 (60,6)	30 (55,56) 24 (44,44)	33 (31,13) 73 (68,87)	**0,003**
Duration of surgery in minutes, median (IQR)		115 (58,5) †	126,5 (64,25) †	111,50 (62,5) †	0,082
Defect size in cm^2^, median (IQR)		9,0 (36,75) †	13 (64,5) †	6,5 (21) †	**0,025**
Mesh size in cm^2^, median (IQR)		450 (120) †	450 (102,75) †	405 (164) †	**0,002**
Transversus Abdominis Release, n (%)		19 (11,9)	7 (12,96)	12 (11,32)	0,761
Intraoperative complications, n (%)		0 (0)	0 (0)	0 (0)	-

† = median + IQR

Additionally, the average mesh size was significantly larger in the drain group compared to the no-drain group (drain: 450 cm^2^ (102,75) †, no-drain: 405 cm^2^ (164) †, *p* = 0,002).

### Outcomes

There were no significant differences regarding the frequency of postoperative complications (drain: 7 (13%), no-Drain: 9 (8,5%), *p* = 0,373), SSO (drain: 7 (13%), no-drain: 5 (4,7%), *p* = 0,108), SSI (drain: 0 (0%), no-drain: 2 (1,9%), *p* = 0,550) and SSOPIs (drain: 4 (7,4%), no-drain: 6 (5,7%), *p* = 0,559) between patients with and without drains. There also were no significant differences regarding the median length of stay in days, recurrence rates and postoperative antibiotic therapies. In both groups Clavien-Dindo score 0 was the most frequently observed (drain: 48 (88,9%), no-drain: 96 (90,9%), *p* = 0,738). Detailed results on postoperative complications and interventions are shown in Table 4 and 5.

**Table 4:** Postoperative findings

	Total (*n* = 160)	Drain (*n* = 54)	No-Drain (*n* = 106)	*p value*
Postoperative complications, n (%)	16 (10)	7 (13)	9 (8,5)	0,373
SSO, n (%)	12 (7,5)	7 (13)	5 (4,7)	0,108
SSI, n (%)	2 (1,3)	0 (0)	2 (1,9)	0,550
SSOPI, n (%)	10 (6,3)	4 (7,4)	6 (5,7)	0,559
Length of stay in days, median (IQR)	2 (1) †	2 (1) †	2 (1) †	0,639
Recurrence, n (%)	5 (3,1)	1 (1,9)	4 (3,8)	0,655
Postoperative antibiotic therapy, n (%)	4 (2,5)	2 (3,7)	2 (1,9)	0,604
Postoperative units of erythrocytes, n (%)	2 (1,3)	1 (1,85)	1 (0,94)	1,000
Clavien-Dindo-Score, n (%)				
0	144 (90)	48 (88,9)	96 (90,9)	0,738
1	7 (4,4)	3 (5,6)	4 (3,8)	0,689
2	1 (0,6)	1 (1,9)	0 (0)	0,338
3	7 (4,4)	2 (3,7)	5 (4,7)	1,000
4	1 (0,6)	0 (0)	1 (0,9)	1,000

*†* = *median* + *IQR*

**Table 5:** Description of Postoperative Complications and Interventions

Postoperative Complications	n	Description	SSOPI	Description
DRAIN				
Intestinal fistula	1	Intestinal fistula in the right lower abdomen three days after eTEP	yes	Exploratory laparotomy, lavage, fistula closure, mesh explantation, placement of a new mesh
Seroma	1	Seroma: 2,5 × 1,5 × 1,5 cm	no	Conservative therapy
Hematoma	1	Partially organized hematoma in the abdominal wall 3 × 6 cm	yes	Puncture of 80 ml of bloody fluid
Hematoseroma	1	Hematoseroma: 4 × 5 cm	yes	Puncture of 60 ml of bloody-serous fluid
Hematoseroma	1	Hematoseroma: 5 × 8 cm	no	Conservative therapy
Hb-relevant postoperative bleeding with haematoma	1	Hb drop of six points, extensive hematoma of the abdominal wall	no	Transfusion oft two units of erythrocytes
Postoperative bleeding with hematoma	1	Postoperative swelling, large, epifascial hematoma	yes	Revision with hematoma evacuation
NO DRAIN				
Hematoseroma	1	Hematoseroma three weeks postoperative	yes	Punction of 60 ml of bloody-serous fluid
Sepsis	1	Sepsis because of abdominal wall abscess	yes	Laparotomy, resection colon descendent, catecholamine therapy
Abscess	1	Periumbilical abscess two weeks postoperative	yes	Revision with VAC system
Early recurrence	1	Early recurrence with incarceration	yes	Revision with new mesh
Hematoma	1	Hb drop of three points, extensive preperitoneal hematoma	yes	Revision with hematoma evacuation, transfusion of erythrocytes
Persistent pain	1	Persistent pain four weeks postoperative	no	-
Superficial bleeding	1	Superficial bleeding on the operation side	yes	Direct suture
Hematoma	1	Organized hematoma: 5 × 7 cm	no	Conservative therapy
Persistent pain	1	Persistent pain four weeks postoperative	no	-

In order to adjust for potential confounding, we performed.

1. logistic regressions of SSO, SSOPI, Clavien-Dindo score of three or higher, and any postoperative complications on operation method, mesh size, and drain,
2. a linear regression of duration of surgery on defect size, reception of TAR, anticoagulation usage, operation method, mesh size, and drain and
3. a Poisson regression of number days of hospitalisation beyond the first day on defect size, reception of TAR, anticoagulation usage, operation method, mesh size, and drain.

None of regressions differed in terms of significance of the effect of drain: length of stay (*p* = 0.140), duration of surgery (*p* = 0.504), any postoperative complication (*p* = 0.442), Clavien-Dindo score of three or higher (*p* = 0.591), SSOPI (*p* = 0.754), SSO (*p* = 0.112).

### Subgroup analysis: Laparoscopic eTEP VS robotic eTEP

Patients who underwent robotic eTEP required drains significantly more often than those who were operated laparoscopically (rob: 30 (47,6%), lap: 24 (24,7%), *p* = 0,003). Furthermore, patients who underwent robotic surgery showed significantly larger defects compared to those who had laparoscopic surgery (rob: 28 cm^2^ (72)†, lap: 6 cm^2^ (9,87)†, p < 0,001). A significantly higher proportion of patients in the robotic eTEP group received TAR compared to those in the laparoscopic eTEP group (rob: 14 (22,2%), lap: 5 (5,2%), *p* = 0,001). Further results from the subgroup analysis can be found in Table 6.

**Table 6:** Subgroup analysis: Laparoscopic eTEP VS robotic eTEP

	Robotic eTEP (*n* = 63)	Laparoscopic eTEP (*n* = 97)	*p value*
Age in years, mean (SD)	54,48 (± 13,76) *	52,19 (± 13,22) *	0,294
BMI, median (IQR)	29 (9) †	31 (9) †	0,159
ASA-Score, n (%)			
1	8 (12,7)	6 (6,2)	0,152
2	38 (60,3)	64 (66)	0,467
3	17 (27)	27 (27,8)	0,906
Preexisting condition: cardiovascular system, n (%)	29 (46)	47 (48,5)	0,764
Preexisting condition: Lung, n (%)	9 (14,3)	9 (9,3)	0,327
Obesity, n (%)	28 (44,4)	52 (53,6)	0,257
Diabetes, n (%)	9 (14,3)	11 (11,3)	0,582
Smoking, n (%)	16 (25,4)	22 (22,7)	0,693
Liver insufficiency, n (%)	1 (1,6)	0 (0)	0,394
Use of anticoagulants, n (%)	8 (12,7)	14 (14,4)	0,756
Duration of surgery in minutes, median (IQR)	120 (61) †	108 (60,5) †	**0,020**
Defect size in cm^2^, median (IQR)	28 (72) †	6 (9,87) †	** < 0,001**
Mesh size in cm^2^, median (IQR)	420 (190) †	450 (150) †	**0,001**
Transversus Abdominis Release, n (%)	14 (22,2)	5 (5,2)	**0,001**
Drain n (%)	30 (47,6)	24 (24,7)	**0,003**

* = mean + SD; † = median + IQR

### *Subgroup analysis: Defect size* > *10 cm*.^*2*^

76 Patients had a ventral hernia larger than 10 cm^2^. 31 of them received a drain, while 45 did not. There were no significant differences regarding the frequency of postoperative complications (drain: 5 (16,1%), no-Drain: 4 (8,9%), *p* = 0,473), SSO (drain: 5 (16,1%), no-drain: 3 (6,7%), *p* = 0,259), SSI (drain: 0 (0%), no-drain: 1 (2,2%), *p* = 1,000) and SSOPIs (drain: 3 (9,7%), no-drain: 4 (8,9%), *p* = 0,444) between patients with and without drains. Further results from the subgroup analysis can be found in Table 7.

**Table 7:** Subgroup analysis: Defect size > 10 cm^2^

		Total (*n* = 76)	Drain (*n* = 31)	No-Drain (*n* = 45)	*p value*
Postoperative complications, n (%)		9 (11,8)	5 (16,1)	4 (8,9)	0,473
SSO, n (%)		8 (10,5)	5 (16,1)	3 (6,7)	0,259
SSI, n (%)		1 (1,3)	0 (0)	1 (2,2)	1,000
SSOPI, n (%)		7 (9,2)	3 (9,7)	4 (8,9)	0,444
Length of stay in days, median (IQR)		2 (1)	2 (1)	2 (1)	0,817
Recurrence, n (%)		1 (1,3)	0 (0)	1 (2,2)	1,000
Postoperative antibiotic therapy, n (%)		3 (3,9)	2 (6,5)	1 (2,2)	0,563
Clavien-Dindo-Score, n (%)					
0		68 (89,5)	27 (87,1)	41 (91,1)	0,709
1		4 (5,3)	3 (9,7)	1 (2,2)	0,298
2		0 (0)	0 (0)	0 (0)	-
3		3 (3,9)	1 (3,2)	2 (4,4)	1,000
4		1 (1,3)	0 (0)	1 (2,2)	1,000

† = median + IQR.

## Discussion

The traditional surgical approach promotes the use of wound drain following ventral hernioplasties to prevent postoperative complications such as haematomas, seromas, wound dehiscence, and infections, thereby safeguarding the surgical outcome [3]. However, the decision regarding the use of drains in ventral hernia surgery often relies on the personal preferences and experiences of the surgeon due to a lack of evidence or clear consensus guidelines [19]. The theory suggests that drains can help to remove excess fluid collections and thus reduce the risk of wound-specific complications such as seromas [20]. On the other hand is also conceivable that the use of drains may not provide the anticipated benefits and that these could potentially be overshadowed by the increased risk of drain-associated complications, such as postoperative wound and mesh infections [21].

In this study, we compared patients who received a drain during their eTEP hernioplasty with those who did not, focusing on significant differences in demographic and clinical data, as well as the incidence of postoperative complications. The comparison groups were overall similar, with no significant differences observed in demographic data, the prevalence of various comorbidities or the type of hernia (primary or incisional).

Epigastric (EHS-M2) and umbilical (EHS-M3) hernias were the most common types observed in our cohort, together representing just over 80% of all hernias. This finding is consistent with other studies on this topic; for instance, in the retrospective analysis by Lima et al. [19].

With 60% of the participants undergoing laparoscopic surgery, this method was the most common approach. This trend may be observed because our centre has only been using a surgical robot since 2022, resulting in a limited number of robotic eTEPs performed by the time of the analysis.

Furthermore, we observed that patients in the drain group had significantly larger hernia defects compared to those who did not receive a drain (13 cm^2^ versus 6.5 cm^2^, *p* = 0,025). These findings are consistent with results from other studies: in the registry study by Sahm et al., patients who received a drain also showed larger defects than those who did not [22]. Similar findings were observed in the study by Indrees et al., which indicated that patients with hernias larger than 10 cm were more likely to receive drains [23]. Larger defects often represent a more complex surgical challenge, potentially necessitating the use of drains [21]. This may provide an explanation for our results. Given the significant difference in defect size between the study groups and recognizing that the defect size may be an important factor influencing the use of drains we conducted a subgroup analysis including patients with hernia defects larger than 10 cm^2^ The majority (60%) did not receive a drain suggesting that defect size alone was not a factor for the surgeons’ decision to use a drain. The subgroup analysis showed no significant differences regarding postoperative complications, SSOs or SSIs. Overall, the rate of postoperative complications in patients with defects > 10 cm^2^ was similar (11,8%) as in our main analysis including patients with all defect sizes (10%).

Overall, our main analysis found no significant differences in the incidence of postoperative complications, SSOs or SSIs, nor in the length of hospital stay between eTEP patients with and without drains. However, the majority of SSOs observed in the drain group -approximately 86%- were haematomas or haematoseromas, casting doubt on the assumption that drains effectively reduce or prevent such complications. Similar findings were reported in the study by Arora et al. [6]: As in our findings, the authors reported no significant differences in the incidence of SSOs or SSIs between patients with and without drains. A meta-analysis by Marcolin et al., that examined data from over 1700 eTEP patients, also found no significant differences in SSO rates between patients with and without drains [1]. The only notable difference was that seromas occurred significantly less frequently in the drain group compared to the no-drain group. However, the authors included both, patients receiving minimally invasive surgery but also patients with open repair of ventral hernias, constricting the transferability of results on minimally invasive hernioplasties.

In our analysis, there was no significant difference in incidence of SSOPI between patients with and without drains. When calculating SSOPI rates out of all patients with SSOs and SSIs, they were found to be nearly similar: drain group: 57%, no-drain group: 67%. Similarly, Marcolin et al. reported no significant differences in SSOPI rates between the comparison groups [1]. In our study, SSOPIs included two cases of mesh explanations: one patient in the drain group developed a small bowel fistula three days postoperatively. Another patient in the no-drain group required revision surgery two days postoperatively due to early recurrence with incarceration.

The overall incidence of SSIs in our patient cohort was low, with only two patients developing an SSI, representing a rate of 1.25%. This is comparable to SSI rates reported in other studies on laparoscopic ventral hernioplasties. For instance, a meta-analysis by Arita et al. in 2014, which included 20 studies, found an SSI rate of 1.6% for laparoscopically repaired ventral and incisional hernias [24]. In our analysis, both patients with SSIs were in the no-drain group. No SSIs were observed in the drain group, indicating that in our study, drains did not result in drain-associated infections.

Similar findings have been observed in other studies. Notably, studies analysing data from the Americas Hernia Society Quality Collaborative (AHSQC) concluded that the use of drains did not lead to an increased rate of SSIs [15, 21].

However, there are also studies suggesting that the use of drains may elevate the risk of SSIs, by allowing retrograde bacterial migration through external or luminal contamination [25, 26]. This is particularly relevant in hernia surgery due to potential infection risk for the implanted mesh located near the drain. A meta-analysis by Mohamedahmed et al., which included 2,468 patients, found significantly higher SSI rates in the drain group [3]. Moreover, the authors did not find any evidence of a preventive effect of drains on seroma formation. However, it is important to note that this study also included patients with open repair of ventral hernias which may be an influencing factor on the occurrence of SSIs and limit the generalizability of the findings on patients who received laparoscopic eTEP.

Overall, even the most recent studies offer inconsistent results regarding whether drains in ventral hernioplasties prevent postoperative complications or represent a potential risk factor for SSIs.

The length of hospital stay did not differ significantly in our study between patients with and without drains. The median duration of inpatient stay was two days in both groups. These findings are not consistent with those of other studies, which reported significantly longer hospital stays for hernioplasty patients with drains [15, 19, 20, 27]. Miller et al. suggested several possible reasons for this discrepancy: the drain itself may have delayed discharge, the individual practices of the responsible surgeons could have played a role or patients with drains might have undergone potentially more complex procedures that necessitated a longer recovery time [15]. However, their registry-based analysis did not clarify factors influencing surgeons' decisions to use drains or provide detailed clinical information on postoperative complications and recovery.

Possible explanations for the similar length of hospital stay among patients with and without drains in our study could include the discharge management protocols at our clinic, which in exceptional cases allowed patients with drains to return home. In such cases, the drains were removed during an outpatient follow-up appointment shortly thereafter. Another reason might be that drains were removed early enough to ensure that the length of stay for affected patients did not exceed that of those without drains.

In our study, we observed that patients who underwent robotic surgery received drains significantly more often (30 out of 63 patients, 47,6%, *p* = 0,003) compared to those who had laparoscopic surgery (24 out of 97 patients, 24,7%, *p* = 0,003). To investigate potential causes for this difference, we conducted a subgroup analysis comparing laparoscopically operated patients with those who underwent robotic surgery to identify significant differences. We particularly focused on factors that might have been associated with the use of drains, such as hernia size, risk factors including pre-existing conditions or the use of anticoagulants, as well as the performance of a TAR in addition to eTEP surgery. Indeed, we found that patients undergoing robotic eTEP had significantly larger defect sizes than those undergoing laparoscopic eTEP (rob: 28 cm^2^, lap: 6 cm^2^, p < 0,001), which could have contributed to the surgeon’s decision to use a drain, as larger defects often cause a larger wound surface, potentially resulting in more diffuse bleeding tendency. The larger defect size may also explain why the proportion of patients receiving a TAR was significantly higher in the robotic-eTEP group compared to the laparoscopic-eTEP group (rob: 22,2%, lap: 5,2%, *p* = 0,001), as TARs are primarily indicated for large and complex defects [17]. Performing a TAR in addition to eTEP requires additional time, this also accounts for the significantly longer operative duration in the robotic-eTEP group (rob: 120 min, lap: 108 min, *p* = 0,020). In summary, these significant differences between the robotic- and laparoscopic-eTEP group may provide a possible explanation for the higher rate of drains in the robotic-eTEP group. This, in turn, would elucidate our observation that patients in the drain group significantly more often received robotic surgery compared to those in the no-drain group.

To detect and adjust for potential confounding, we conducted additional subgroup analyses and multivariable regressions. The subgroups included patients with primary abdominal wall hernias, patients with incisional abdominal wall hernias, patients who received a TAR and patients who did not. Most of the subgroup analyses showed no significant differences regarding postoperative complications, recurrences or length of hospital stay between patients with and without drains. Only the subgroup analysis of patients who did not receive a TAR showed a significant higher rate of SSOs in the drain group compared to the no-drain group (12,8% versus 2,1%; *p* = 0,017). This finding indicates that drains were ineffective in preventing surgical site occurrences (SSOs) in patients with non-complex hernias that did not require TAR, suggesting that the use of drains may be unnecessary in such cases.

### Strengths and limitations

Our study is based on a comprehensive monocentric dataset of 160 cases. The monocentric design ensures consistent standards in patient recruitment, treatment and follow-up, improving the overall data quality in our study, minimising variability. All eTEP procedures were performed by the same surgeon, further minimising variability, particularly regarding the decision to use drains.

The comparison groups were similar in demographic characteristics and risk factors.

However, this study also has some limitations that must be considered when interpreting our results due to the retrospective and non-randomised design of the study: The low number of SSIs, SSOs, SSOPIs and complications in general limited our possibilities to account for potential confounding. The number of observed SSIs and recurrences were so small that we lack the statistical power necessary to detect a meaningful difference. Lastly, the retrospective design of our study was susceptible to information bias and resulted in incomplete datasets in some cases, leading to exclusions of certain patients.

### Conclusion

In our main analysis, no significant differences were observed between patients with and without drains following hernioplasty using the eTEP technique concerning length of hospital stay or the occurrence of postoperative complications, SSOs and SSIs. Our data raises the question if drains are indeed beneficial in ventral hernioplasties and encourages a consideration of whether the use of drains after hernioplasties performed via the eTEP technique may pose more risks than benefits. Particularly in patients with non-complex hernias who did not require TAR, the use of drains did not effectively prevent surgical site occurrences (SSOs). In fact, patients with drains showed a significantly higher rate of SSOs compared to those without drains.

SSIs were overall rare in our patient cohort but occurred exclusively in the no-drain group. Thus, our data does not support that drains lead to an increased incidence of infections. In this context, further research is necessary to provide greater clarity regarding the heterogeneous findings in the literature on this topic.

In summary, this study does not support nor refute the conclusion that wound drains effectively prevent postoperative complications such as seromas or haematomas and should be routinely employed in minimally invasive hernioplasty procedures. Additional studies are required to evaluate both the benefits and risks associated with drains concerning the occurrence of postoperative complications following hernioplasties.

## Supplementary Information

Below is the link to the electronic supplementary material.

Supplementary file1 (DOCX 35 kb)

## Data Availability

No datasets were generated or analysed during the current study.

## Abbreviations

eTEP: Extended-totally-extraperitoneal Hernioplasty
SSI: Surgical Site Infections
SSO: Surgical Site Occurrences
TAR: Transversus Abdominis Release
IPOM: Intraperitoneal Onlay Mesh
TAPP: Transabdominal Preperitoneal Hernioplasty
BMI: Body Mass Index
COPD: Chronic Obstructive Lung Disease
ASA: American-Society-of-Anaesthesiologists
EHS: European-Hernia-Society
SSOPI: Surgical Site Occurrences Requiring Procedural Interventions
AHSQC: Americas Hernia Society Quality Collaborative
