# Supplementary material for: To drain or not to drain in minimal invasive ventral hernia surgery
Source: Langenbecks Arch Surg. 2025 Mar 11;410(1):97. doi: 10.1007/s00423-025-03668-x (PMC11897075; doi:10.1007/s00423-025-03668-x)
Supplement: Supplementary file 1 — Supplementary file1 (DOCX 35 kb) [file 423_2025_3668_MOESM1_ESM.docx]

Supplementary Material

Subgroup analyses

Furthermore, subgroup analyses were performed for patients with primary abdominal wall hernias, for patients with incisional abdominal wall hernias, for patients that received a TAR and for patients that did not receive a TAR. Most of the subgroup analyses showed no significant differences regarding postoperative complications, recurrences or length of hospital stay between patients with and without drainage. Only the subgroup of patients who did not receive TAR showed a significant higher rate of SSOs in the drain group compared to the no-drain group (drain: 6 (12,8%), no-drain (2 (2,1%), p = 0,017). Additional details are shown in Table 8-11.

Table 8 Subgroup analysis: Primary Hernias

|  | | | | | |  |
| --- | --- | --- | --- | --- | --- | --- |
|  |  | | **Total**  (n = 104) | **Drain**  (n = 33) | **No-Drain**  (n = 71) | ***p value*** |
| **Postoperative complications,**  **n (%)** | | 6 (5,8) | | 2 (6,1) | 4 (5,6) | 1,000 |
| **SSO, n (%)** | | 4 (3,8) | | 2 (6,1) | 2 (2,8) | 0,590 |
| **SSI, n (%)** | | 1 (1) | | 0 (0) | 1 (1,4) | 1,000 |
| **SSOPI, n (%)** | | 4 (3,8) | | 1 (3) | 3 (4,2) | 1,000 |
| **Length of stay,**  **median (IQR)** | | 2 (1) † | | 3 (1) † | 2 (1) † | 0,387 |
| **Recurrence, n (%)** | | 4 (3,8) | | 1 (3) | 3 (4,2) | 1,000 |
| **Postoperative**  **Antibiotic therapy, n (%)** | | 1 (1) | | 0 (0) | 1 (1,4) | 1,000 |
| **Clavien-Dindo-Score, n (%)**  0  1  2  3  4 | | 96 (92,3)  3 (2,9)  1 (1,0)  4 (3,8)  0 (0) | | 30 (90,9)  2 (6,1)  1 (3)  0 (0)  0 (0) | 66 (93)  1 (1,4)  0 (0)  4 (5,6)  0 (0) | 0,706  0,239  0,317  0,304  - |
| †= median+IQR | | | | | | |
|  | |  | |  |  |  |

Table 9 Subgroup analysis: Incisional Hernias

|  | | | | | |  | |
| --- | --- | --- | --- | --- | --- | --- | --- |
|  |  | | **Total**  (n = 56) | **Drain**  (n = 21) | **No-Drain**  (n = 35) | ***p value*** |  |
| **Postoperative complications,**  **n (%)** | | 10 (17,9) | | 5 (23,8) | 5 (14,3) | 0,476 | |
| **SSO, n (%)** | | 8 (14,3) | | 5 (23,8) | 3 (8,6) | 0,136 | |
| **SSI, n (%)** | | 1 (1,8) | | 0 (0) | 1 (2,9) | 1,000 | |
| **SSOPI, n (%)** | | 6 (10,7) | | 3 (14,3) | 3 (8,6) | 1,000 | |
| **Length of stay in days,**  **median (IQR)** | | 2 (1) † | | 2 (1) † | 3 (1) † | 0,598 | |
| **Recurrence, n (%)** | | 1 (1,8) | | 0 (0) | 1 (2,9) | 1,000 | |
| **Postoperative antibiotic therapy, n (%)** | | 3 (5,4) | | 2 (9,5) | 1 (2,9) | 0,549 | |
| **Clavien-Dindo-Score, n (%)**  0  1  2  3  4 | | 48 (85,7)  4 (7,1)  0 (0)  3 (5,4)  1 (1,8) | | 18 (85,7)  1 (4,8)  0 (0)  2 (9,5)  0 (0) | 30 (85,7)  3 (8,6)  0 (0)  1 (2,9)  1 (2,9) | 1,000  1,000  -  0,549  1,000 | |
| †= median+IQR | | | | | | | |
|  | |  | |  |  |  | |

Table 10 Subgroup analysis: Patients who received TAR

|  | | | | | |  |
| --- | --- | --- | --- | --- | --- | --- |
|  |  | | **Total**  (n = 19) | **Drain**  (n = 7) | **No-Drain**  (n = 12) | ***p value*** |
| **Postoperative complications, n (%)** | | 5 (26,3) | | 1 (14,3) | 4 (33,3) | 0,603 |
| **SSO, n (%)** | | 4 (21,1) | | 1 (14,3) | 3 (25) | 1,000 |
| **SSI, n (%)** | | 1 (5,3) | | 0 (0) | 1 (8,3) | 1,000 |
| **SSOPI, n (%)** | | 5 (26,3) | | 1 (14,3) | 4 (33,3) | 0,603 |
| **Length of stay in days,**  **median (IQR)** | | 3 (2) † | | 2 (2) † | 3 (2) † | 0,382 |
| **Recurrence, n (%)** | | 0 (0) | | 0 (0) | 0 (0) | - |
| **Postoperative**  **antibiotic therapy, n (%)** | | 2 (10,5) | | 1 (14,3) | 1 (8,3) | 1,000 |
| **Clavien-Dindo-Score, n (%)**  0  1  2  3  4 | | 14 (73,7)  2 (10,5)  0 (0)  2 (10,5)  1 (5,3) | | 6 (85,7)  1 (14,3)  0 (0)  0 (0)  0 (0) | 8 (66,7)  1 (8,3)  0 (0)  2 (16,7)  1 (8,3) | 0,603  1,000  -  0,509  1,000 |
| †= median+IQR | |  | |  |  |  |
|  | |  | |  |  |  |

Table 11 Subgroup analysis: Patients who did not receive TAR

|  | | | | |  |
| --- | --- | --- | --- | --- | --- |
|  |  | **Total**  (n = 141) | **Drain**  (n = 47) | **No-Drain**  (n = 94) | ***p value*** |
| **Postoperative complications, n (%)** | | 11 (7,8) | 6 (12,8) | 5 (5,3) | 0,180 |
| **SSO, n (%)** | | 8 (5,7) | 6 (12,8) | 2 (2,1) | **0,017** |
| **SSI, n (%)** | | 1 (0,7) | 0 (0) | 1 (1,1) | 1,000 |
| **SSOPI, n (%)** | | 5 (3,5) | 3 (6,4) | 2 (2,1) | 1,000 |
| **Length of stay in days,**  **median (IQR)** | | 2 (1) † | 2 (1) † | 2 (1) † | 0,481 |
| **Recurrence, n (%)** | | 5 (3,5) | 1 (2,1) | 4 (4,3) | 0,657 |
| **Postoperative**  **antibiotic therapy, n (%)** | | 2 (1,4) | 1 (2,1) | 1 (1,1) | 1,000 |
| **Clavien-Dindo-Score, n (%)**  0  1  2  3  4 | | 130 (92,2)  5 (3,5)  1 (0,7)  5 (3,5)  0 (0) | 42 (89,4)  2 (4,3)  1 (2,1)  2 (4,3)  0 (0) | 88 (93,6)  3 (3,2)  0 (0)  3 (3,2)  0 (0) | 0,506  1,000  0,333  1,000  - |
| †= median+IQR | |  |  |  |  |
|  | |  |  |  |  |
